# *In Vivo* Imaging with Fluorescent Smart Probes to Assess Treatment Strategies for Acute Pancreatitis

**DOI:** 10.1371/journal.pone.0055959

**Published:** 2013-02-11

**Authors:** Abhiruchi Agarwal, Andreas Boettcher, Rainer Kneuer, Farid Sari-Sarraf, Adriana Donovan, Julian Woelcke, Oliver Simic, Trixi Brandl, Thomas Krucker

**Affiliations:** 1 Novartis Institute of BioMedical Research, Cambridge, Massachusetts, United States of America; 2 Novartis Institutes for BioMedical Research, Basel, Switzerland; 3 Novartis Institutes of BioMedical Research, Emeryville, California, United States of America; Southern Medical University, China

## Abstract

**Background and Aims:**

Endoprotease activation is a key step in acute pancreatitis and early inhibition of these enzymes may protect from organ damage. *In vivo* models commonly used to evaluate protease inhibitors require animal sacrifice and therefore limit the assessment of dynamic processes. Here, we established a non-invasive fluorescence imaging-based biomarker assay to assess real-time protease inhibition and disease progression in a preclinical model of experimental pancreatitis.

**Methods:**

Edema development and trypsin activation were imaged in a rat caerulein-injection pancreatitis model. A fluorescent “smart” probe, selectively activated by trypsin, was synthesized by labeling with Cy5.5 of a pegylated poly-L-lysine copolymer. Following injection of the probe, trypsin activation was monitored in the presence or absence of inhibitors by *in vivo* and *ex vivo* imaging.

**Results:**

We established the trypsin-selectivity of the fluorescent probe *in vitro* using a panel of endopeptidases and specific inhibitor. *In vivo*, the probe accumulated in the liver and a region attributed to the pancreas by necropsy. A dose dependent decrease of total pancreatic fluorescence signal occurred upon administration of known trypsin inhibitors. The fluorescence-based method was a better predictor of trypsin inhibition than pancreatic to body weight ratio.

**Conclusions:**

We established a fluorescence imaging assay to access trypsin inhibition in real-time *in vivo.* This method is more sensitive and dynamic than classic tissue sample readouts and could be applied to preclinically optimize trypsin inhibitors towards intrapancreatic target inhibition.

## Introduction

Acute pancreatitis is a serious condition characterized by inflammation, fibrosis and endocrine and exocrine dysfunction of the pancreas. It has a high incidence rate [1], [2] and a mortality of up to 40% [1], [3], [4] in the US. Genetic and environmental factors can lead to an inappropriate activation of trypsin proteases, lipases and other zymogens causing direct pancreatic injury, which in turn triggers an inflammatory immune response.

There is growing evidence that genetic variants underlie susceptibility to acute pancreatitis. Hereditary pancreatitis is generally described as an autosomal dominant gain-of-function disorder related to mutations in the cationic trypsinogen gene *PRSS1*, which has an 80% penetrance. Mutations in this gene promote premature cleavage of trypsinogen to active trypsin in the pancreatic acinar cells, which in turn causes pancreatic autodigestion [5], [6], [7]. In addition, trypsinogen copy number variants (duplications and triplications) appear to be associated with idiopathic pancreatitis in some populations [8]. Moreover, loss-of-function mutations in the gene of the endogenous trypsin inhibitor Kazal type 1 (SPINK1) have been reported to be associated with pancreatitis [9]. SPINK1 is important in limiting ongoing trypsin activity in the pancreatic acinar cells after the onset of an acute inflammatory reaction. Studies in SPINK3 (mouse ortholog of human SPINK1) k.o. mice suggest that the *Spink* gene plays an essential role in the maintenance of acinar cells [10].

Protease activation targeting trypsinogen or other zymogens within the acinar cells of the pancreas are considered to be early events in the onset of acute pancreatitis [11], [12]. This strongly enhanced intracellular proteolytic activity results in cell injury and triggers an inflammatory response. Recent investigation of pathophysiologic markers indicates trypsinogen and other pancreatic proteases have close correlation to disease severity [4]. Trypsin activation is believed to be a very early and pivotal step in the onset of the disease; therefore, trypsin inhibition has to be achieved very early in the progression of the disease. In developing drugs for acute pancreatitis, screening of compounds that are direct trypsin inhibitors would be useful.

In experimental *in vivo* models, drug efficacy is examined classically by anatomical/histological changes in the pancreas that necessitate animal sacrifice, and thus making the observation of dynamic and disease-relevant processes in the course of the experiment very difficult if not impossible. Understanding the dynamics of intrapancreatic trypsin activity, the correlation to intrapancreatic edema formation, and the time course of both readouts could benefit the understanding of potential disease mechanisms and greatly enhance preclinical optimization of inhibitors of trypsin as potential drugs for the treatment of acute pancreatitis.

*In vivo* optical imaging is an easy to use technique with the potential of studying molecular targets inside the body of a living animal. Optical imaging can be adapted to visualize and quantitate the progression of a disease, the effects of drug candidates on the target tissue, the pharmacokinetic behavior of drug candidates, and the development of biomarkers indicative of disease and treatment outcomes. This method benefits from the development of activatable or “smart” fluorescent probes that emit signal upon interaction with the target [13]. Activatable probes are made of one or more different fluorophores, which are joined very closely to each other by an enzyme-specific peptide linker. Due to close proximity, the fluorophores are quenched. Therefore, activatable or “smart” probes, when intact, show little to no fluorescence upon excitation. Upon introduction of the specific enzyme and cleavage of the peptide linker, the fluorophores separate from each other and the fluorescence can then be detected. Activatable probes benefit from low background signal and higher contrast and detection sensitivity compared to traditional (always “on”) fluorescent probes. “Activation” effect not only minimizes or removes the high background signal obtained from traditional imaging techniques, but also enables accurate determination of the specific molecular target or function [14].

The work presented here introduces for the first time a non-invasive technique to track the activity of trypsin/protease inhibitor in rat pancreas of an experimental model of caerulein-injection induced pancreatitis, using molecular optical imaging and an activatable reporter. The aim of the present study was to establish a mode-of-action biomarker assay for trypsin activity in rat pancreas of an established preclinical model of experimental pancreatitis to characterize protease inhibitors using non-invasive molecular optical imaging. Such a model can be applied to preclinically optimize trypsin inhibitors in the target tissue.

## Methods

### 1 Ethics Statement

Animal husbandry for this study was provided by Laboratory Animal Services of Novartis Institutes for Biomedical Research (NIBR). These animal experiments were approved by NIBR Animal Care and Use Committee (IACUC), approved protocol number 09IMG096. Rats were handled according to IACUC guidelines and all efforts were made to minimize animal suffering.

### 2 Probe Preparation

Initially the Cy5.5 labeled endoprotease-activatable probe mPEG-PL-Cy5.5 was prepared as described by Weissleder *et al* [15]. Briefly, multiple methoxypolyethylene glycol (mPEG) chains (5 kDa) were covalently attached to poly-L-lysine (PL, 20–30 kD) in bicarbonate buffer (0.1 M). According to NMR analysis approximately 28% of the initially available lysine ε-amino groups were pegylated. The resulting copolymer (calculated average molecular weight 195 kD) was then further modified with the succinimidyl ester of Cy5.5 and the loading efficiency of Cy5.5 was determined by UV/Vis measurements. The local density of fluorochrome was sufficiently high for auto quenching to occur.

All enzyme assays were conducted in 384-well plates with a final substrate ( = mPEG-PL-Cy5.5 probe) concentration of 0.2 µM diluted in the appropriate assay buffer in a final assay volume of 25 µl. Enzyme and, in cases where applied, SPINK-1 (final assay concentration: 1 µM) were pre-incubated for one hour at room temperature. Subsequently substrate was added and incubated for 3 h at 37°C prior to measurement. All enzymes were either commercially obtained or prepared as described previously [16], [17], [18], [19].

Enzyme, SPINK-1 and substrate solutions were transferred to 384-well plates by means of a CyBi Well pipettor (CyBio, Jena, Germany). Plate measurements were conducted by the means of a Safire2 microplate reader (TECAN, Maennedorf, Switzerland) and wavelengths of 680 nm and 700 nm were taken for fluorescence excitation and emission acquisition, respectively. The bandwidths were set to 10 nm in both the excitation and the emission path. The fluorescence in each well was excited by three flashes per measurement.

### 3 Trypsin-inhibitor Protease Selectivity

The three drug-like compounds tested in the optical molecular imaging model were characterized for selectivity against a panel of recombinant proteases available at Novartis. The biochemical enzyme activity assays were based on the cleavage of peptidic substrates that are fluorescently labeled. In case of rhodamine-110 label fluorescence intensity and in case of PT14 label fluorescence lifetime was used as readout. The effect of the compound on the enzymatic activity was obtained from the linear part of the substrate cleavage progress curve at substrate concentrations well below K_M_, typically at 1 µM. All enzyme assays were conducted in 384-well plates with a final assay volume of 25 µl. Enzyme and compound (11 different concentrations from 100 µM to 0.0003 µM) were pre-incubated for one hour at room temperature (RT). Subsequently substrate was added and incubated for 1 h at RT prior to measurement.

### 4 Animal Model

280–320 g female Sprague Dawley rats were received from Charles Rivers Laboratory. Animals were maintained in a facility with a controlled temperature (21–24°C) and lighting (12∶12 h light: dark cycle). Animals were placed on a chlorophyll-free diet (Harlan TK2916) 10 days prior and for the duration of the imaging study to reduce the amount of autofluorescence and had free access to drinking water. One day prior to the experiment, animals were shaved at the abdomen for imaging studies. The pancreatitis model was induced by hyperstimulation with the cholecystokinin (CCK) analog caerulein (Sigma-Aldrich, St. Louis, MO). For Camostat studies, animals were dosed with a solution of Camostat in buffered saline at 300 mg/kg or buffered saline via the oral route 24 hours before and again 1 hour prior to the hyperstimulation with caerulein [20], [21]. For studies with Novartis166 and Novartis848, varying concentrations, in a solution, were administered intraperitonealy (IP) only once, one hour before caerulein administration. Self-quenched protease activatable mPEG-PL-Cy5.5 probe was administered to all animals via the tail vein at a dose of 80 nmol/kg 40 minutes prior to the start of caerulein administration for measuring the amount of activated trypsin in the pancreas. Animals were administered 3 hourly injections of caerulein subcutaneously (SC) at a dose of 50 µg/kg. Animals were euthanized by CO_2_ inhalation one hour after the last caerulein dose and pancreas were collected.

### 5 In Vivo Optical Imaging Studies

In vivo optical imaging studies were conducted using a spectral imaging system, Maestro (Perkin Elmer, Boston, MA). The tunable filter was automatically stepped in 10-nm increments over a 250 nm bandwidth while the camera captured images at each wavelength with constant exposure. Animals were briefly anesthetized using 1–3% isoflurane in 100% oxygen, which was delivered through a nose cone within the imaging chamber and placed inside the imaging system. Animals were imaged before the start of the study for background/autofluorescence, and at 20 minutes after the mPEG-PL-Cy5.5 probe administration for basal increase in fluorescence due to probe administration. Images were acquired 45 minutes after each caerulein administration. One hour after the last caerulein, animals were euthanized by CO_2_ inhalation and the skin over the abdominal cavity was removed. Animals were imaged to assess the source of the fluorescent signal and intensity calculations. Pancreas were excised, weighed, and imaged for total intensity, edema and trypsin concentration assessment.

### 6 Assessment of Trypsin Concentration in Pancreas

The pancreas was removed from the euthanized animals, weighted and transferred into Dispomix tubes (Miltenyi Biotec, Bergisch Gladbach, Germany). After the addition of ice-cold PBS buffer (1 µl per µg tissue), the pancreas was homogenized with a gentleMACS™ Dissociator (Miltenyi Biotec, Bergisch Gladbach, Germany). The homogenate was centrifuged at 4°C and 20,000 g for 10 min. The supernatant was stored at −20°C. The activity assay was performed at room temperature in 384-well plates in a total assay volume of 20 µl per well. For fluorescence quantification a Safire2 (Tecan, Maennedorf, Switzerland) instrument was used with fluorescence excitation and emission wavelengths of 485 nm and 535 nm, respectively. 10 µl of dilutions of extracts (typical range: 1∶500 to 1∶2,000) were transferred to 384 well assay plates. The reaction was started by the addition of 10 µl of a solution of the fluorogenic trypsin substrate Benzoyl-Gly-Pro-Arg-Rh110-γGlu (#BS-8378.1, Biosynthan, Berlin, Germany). The final substrate concentration was 2 µM. The enzymatic activity in the pancreatic extract was obtained from the linear progression curves and determined after one hour. Signal-to-background (S/B) values within the linear range of the assay signal were selected to calculate the trypsin concentrations of the extract analyzed. In order to distinguish between apparent and specific trypsin activity, the endogenous trypsin inhibitor SPINK-1 was added to the concentrated rat extracts at 10,000 fold its IC_50_ concentration ( = 1 µM). The remaining enzymatic activity, which does not originate from trypsin, was subtracted from the apparent trypsin activity and resulted in the specific trypsin activity. The amount of trypsin was calculated by transforming S/B levels obtained in the activity assay of extract dilutions fulfilling initial enzyme velocity criteria into trypsin concentration values via a calibration curve using recombinant rat trypsin S1-94.

### 7 Data Analysis Imaging Experiments

Each data point was captured as a stack of TIFF images acquired at 10-nm increments and was loaded into a single data structure in the memory, forming a spectral stack with a spectrum at every pixel. Using the spectral imaging software autofluorescence, food, and activated mPEG-PL-Cy5.5 probe spectra were manually selected from the spectral image by selecting appropriate regions. Using available spectral unmixing algorithms the unmixed images of ‘pure’ autofluorescence, and ‘pure’ mPEG-PL-Cy5.5 were created. When appropriately generated, the autofluorescence image should be uniform in intensity regardless of the presence or absence of the dye. The clean images containing only Cy5.5 fluorescent signal were used for further analysis.

#### 7.1 *In vivo* time course

Region of the pancreas and liver were determined and defined from whole body clean images acquired after sacrificing the animals, and used on all corresponding *in vivo* images in the same animal. Fluorescent intensity at various time points from the same animal were normalized by fluorescence intensity determined before the start of the caerulein insult and the change in signal intensity was then plotted as a function of time.

#### *7.2 Ex vivo* correlation

The total fluorescent signal acquired for bare pancreas was plotted against the corresponding edema ratio defined as the ratio of the pancreatic and body weight of each subject in a correlation plot.

## Results

### 8 MPEG-PL-Cy5.5 Probe Characterization

In order to determine trypsin activation in experimental pancreatitis using the caerulein model, we developed a probe that was selective to trypsin activity. To evaluate the mechanism of action, *in vivo*, of trypsin inhibitor drug-like compounds, we prepared a self-quenched trypsin activatable near infrared smart probe.

**Figure 1:**
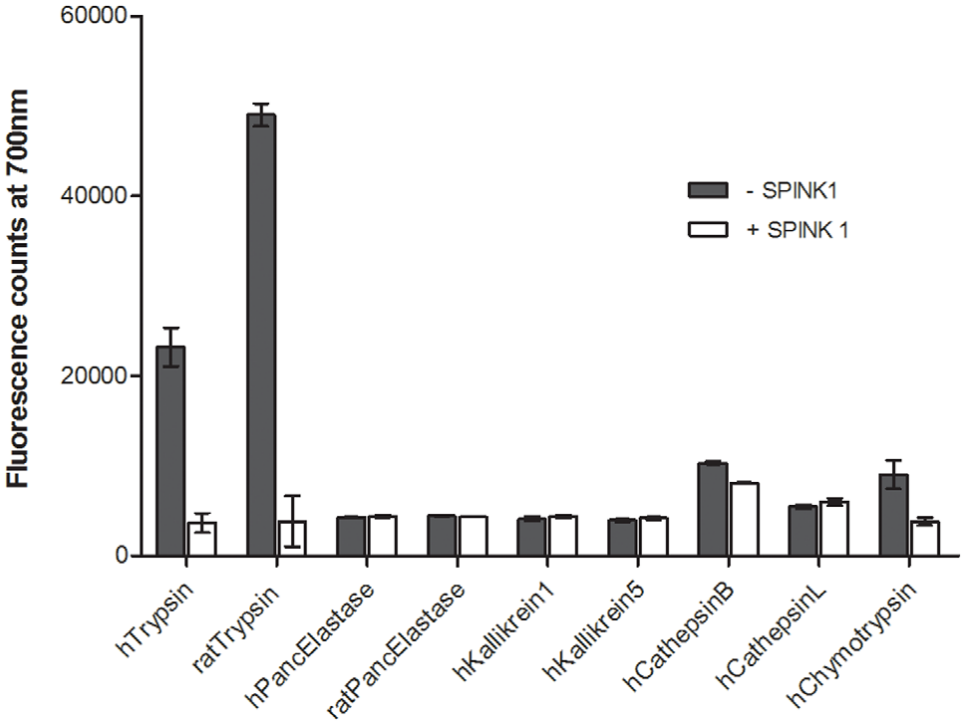
MPEG-PL-Cy5.5 probe characterization. Activation of 0.2 µM of the mPEG-PL-Cy5.5 probe by 100 nM of various pancreatic enzymes in the presence or absence of 1 µM of SPINK1. Probe is specifically activated in the presence of trypsin. Trypsin activity is diminished in the presence of SPINK1. Data represented mean ± SD.

When rat anionic trypsin was added to 1 µM of mPEG-PL-Cy5.5 probe, increase in fluorescence intensity was observed which was positively correlated to the concentration of trypsin enzyme and the time of incubation. To establish trypsin selectivity, mPEG-PL-Cy5.5 probe activation was tested by addition to a panel of endopeptidases that are described to be expressed in the pancreas, including human and rat pancreatic elastase, kallikrein 1, kallikrein 5, chymotrypsin, cathepsin L, and cathepsin B [22]. As shown in figure 1, mPEG-PL-Cy5.5 probe was activated in the presence of trypsin in contrast to other pancreatic enzymes. In the presence of the highly specific trypsin inhibiting protein SPINK1, the protease activation was suppressed to background levels (figure 1). Therefore, it can be concluded that mPEG-PL-Cy5.5 probe was highly trypsin selective.

### 9 In Vivo Monitoring of Trypsin Activation

#### 9.1 Model development

**Figure 2:**
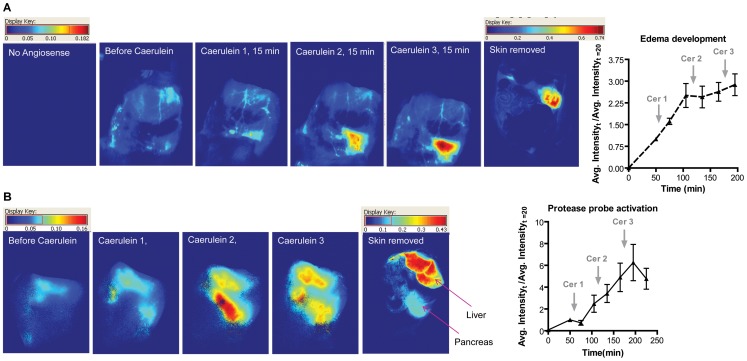
*In vivo* pancreatitis imaging of edema (A) and trypsin activation (B). A). Animals (n = 3) were imaged before and after administering the blood pool agent Angiosense 680. Animals were then administered three subsequent doses of caerulein and imaged at various time points. B). Animals (n = 3) were administered the mPEG-PL-Cy5.5 probe 1 h before first caerulein injection. Data represented mean ± SEM.

The caerulein-injection model is a well-established mechanistic model for experimental pancreatitis. For optical imaging, however, the anatomical location of the pancreas posed a challenge to signal acquisition. Animal weight and age were critical when choosing subjects for optical imaging study. Adult rats were considered the most suitable because of their smaller stomach size and separation of the pancreas and stomach. Upon administration of caerulein in rats, edema development is observed. We administered a blood pool imaging agent Angiosense 680 to confirm our ability to image pancreas non-invasively in rats. Healthy animals that were subjected to repeated doses of caerulein showed increasing accumulation of the blood pool agent Angiosense 680. The graph in figure 2a shows a threefold enhancement in Angiosense 680 signal in the pancreas normalized to signal acquired before caerulein administration. Therefore, for these studies, three caerulein administrations were sufficient.

Establishing the ability to image diseased pancreas in a rat model using optical imaging, we evaluated the time course and severity of trypsin activation in this experimental pancreatitis model using the trypsin activatable mPEG-PL-Cy5.5 probe.

#### 9.2 Trypsin dependent activation of mPEG-PL-Cy5.5 probe

MPEG-PL-Cy5.5 probe was administered to healthy animals and then three hourly repeated caerulein injections were administered to evaluate the time and severity of trypsin activation. MPEG-PL-Cy5.5 probe accumulation was observed in two regions in animals with pancreatitis. The liver was a constantly increasing and significant source of fluorescence attributed to its role in clearance. The other region contributing to enhancement in fluorescent signal was found below the liver attributed to the pancreas after necropsy (figure 2b). A fivefold enhancement in signal intensity was observed compared to the time point from before caerulein administration. Therefore, *in vivo* fluorescent imaging was further used to evaluate the role of trypsin inhibitors in the caerulein mechanistic pancreatitis model. Occasionally, there was signal enhancement in the duodenal region as well.

We compared the mPEG-PL-Cy5.5 probe to a commercially available protease activatable probe Prosense (Perkin Elmer, Waltham, MA). When compared to the in-house trypsin activatable mPEG-PL-Cy5.5 probe, Prosense was found to be unsuitable for use in the experimental pancreatitis model. We believe that due to its large size (∼400 kD), Prosense was unable to extravasate in significant quantities in the pancreas in the given window of three hours. The signal enhancement in the pancreas was barely distinguishable over time in disease animals (data not shown). From the edema imaging study with Angiosense (∼250 kD), it was known that a probe of up to 250 kD should be able to extravasate in the pancreas with caerulein insult. MPEG-PL-Cy5.5 probe (∼200 kD), as expected, showed a fivefold signal enhancement suggesting that the size of the probe is also an important factor in optical imaging of pancreatitis in the rat model. To further characterize the mPEG-PL-Cy5.5 probe, pH sensitivity and intra- vs. extracellular trypsin activation was investigated (see supplemental figure S1 and S2).

#### 9.3 Functional vs. anatomical comparison

**Figure 3:**
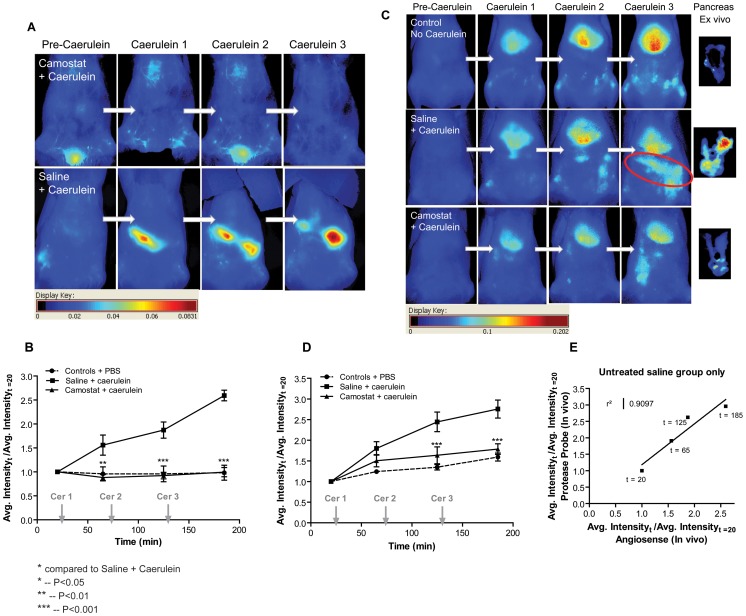
*In vivo* study with trypsin and serine protease activity inhibitor Camostat. A) Animals were administered Camostat at 300 mg/kg orally 24 h and 2 h prior to imaging study. Blood pool fluorescent contrast agent Angiosense was administered intravenously and animals were imaged at different times during a 3 h study with Caerulein induced pancreatitis to assess the development of edema. B) (n = 3) The graph is a quantification of the fluorescent intensity corresponding to the time points in A) and normalized to image obtained prior to caerulein administration. C) Another set of animals was administered the mPEG-PL-Cy5.5 probe to monitor the activity of trypsin in caerulein induced pancreatitis animals. D) (n = 6) The signal in the pancreas was quantified and plotted. Data represented mean ± SEM. E) Untreated saline animal average normalized fluorescent intensity from the Angiosense animals and mPEG-PL-Cy5.5 probe was plotted.

Edema reduction is a prominent outcome of a trypsin inhibitor study in a caerulein model. However, edema reduction gives inadequate information about trypsin inhibition in the pancreas because it is a biomarker not specific to the target. We compared animals pretreated with Camostat (see Table 1 for protease selectivity) at a dose of 300 mg/kg orally to untreated animals. Both sets of animals received three subsequent caerulein doses SC. Animals that did not receive caerulein were used as baseline controls. When imaged using Angiosense 680, there was no signal enhancement as noticed by reduced intensity (figure 3a, b). Healthy control animals did not show any increase in fluorescence in the region of the pancreas. In another study, the same group of animals was imaged using the mPEG-PL-Cy5.5 smart probe. Animals treated with Camostat showed reduced probe activation (figure 3c, d) in comparison to untreated caerulein animals (P<0.001). Camostat treated animals and control animals did not show any significant difference in probe activation after 3 subsequent caerulein administration. All animals indicated high signal intensity in the liver suggesting that the mPEG-PL-Cy5.5 probe is activated by enzymatic activity in disease pancreas. A correlation plot of untreated animals between Angiosense 680 and mPEG-PL-Cy5.5 probe signal intensity in pancreas showed a good correlation between trypsin mediated probe activation and edema accumulation. We were unable to combine the administration of Angiosense 680 and mPEG-PL-Cy5.5 probe in the same animal due to peak broadening of the activated probe signal that bled over into the Angiosense 680 channel.

**Table 1:** Inhibition of tested proteases by compounds Camostat, “Novartis166” and “Novartis848”.

Protease	IC50 value/µM		
	Camostat	Novartis166	Novartis848
h cationic trypsin	0.0028	0.008	0.003
h anionic trypsin	–	0.005	–
rat anionic trypsin S1-94	<0.0003	0.001	0.0001
h thrombin	0.458	39.3	20.5
h factor VIIa	0.531	>100	>100
h factor Xa	6.6	37.6	35.1
h factor XIa	0.008	17.1	0.61
h factor XIIa	8.0	43.0	>100
h plasma kallikrein	0.002	0.133	2.0
h kallikrein 1	72.4	18.8	4.48
h kallikrein 5	0.10	22.0	14.0
h kallikrein 7	41.5	>100	>100
h neutrophil elastase	91.4	48.6	>100
h pancreatic elastase	>50	>100	>100
rat pancreatic elastase		>100	-
h cathepsin G	44.6	61.0	54.6
h proteinase 3	>50	>100	>100
h chymotrypsin	12.17	2.26	59.3
h matriptase	0.0065	>30	>100
h chymase	>50	>100	>100
h cathepsin K	>50	>30	>100
h cathepsin L	>50	>30	>100
h cathepsin S	>50	>30	>100
h cathepsin C	>50	>30	>100
h cathepsin B	>50	-	>100

**Figure 4:**
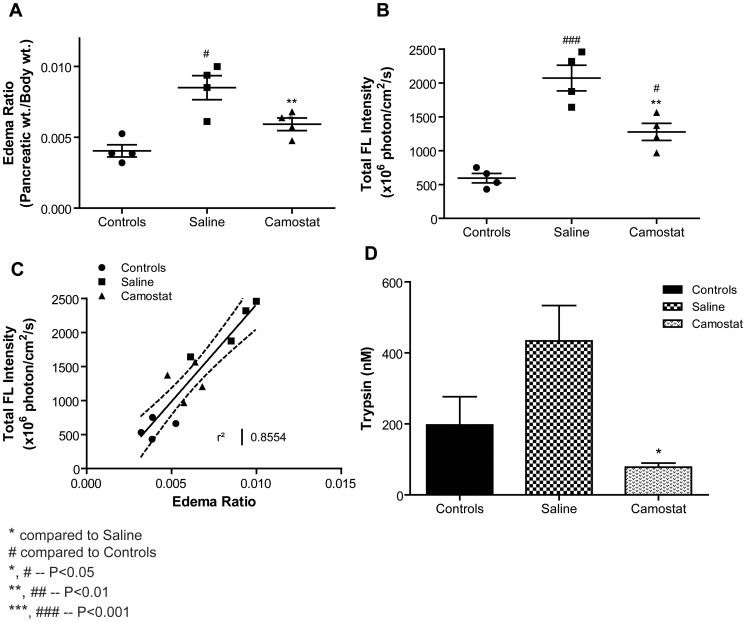
*Ex vivo* analysis of animals receiving the mPEG-PL-Cy5.5 probe in the Camostat study. A) The pancreas from each animal was excised and weighed. Camostat treated animals showed significant reduction in the edema ratio compared to untreated saline animals. B) Total signal intensity of the activated mPEG-PL-Cy5.5 probe in the excised pancreas was quantified. Camostat animals showed a significant reduction in signal intensity compared to untreated saline animals. C) The total signal intensity was plotted against the edema ratio. A positive correlation was observed. D) At the end of the study, pancreas samples were also analyzed for the amount of enzymatically active trypsin. Camostat treated animals showed significant reduction in the amount of active trypsin compared to untreated saline animals. Data represented mean ± SEM.

After the third hour of caerulein, the pancreas was excised from all animals, weighed, and imaged to confirm the *in vivo* observations. Edema ratio was calculated by dividing the weight of the pancreas by the total body weight of the animal. Upon examination of dissected pancreas, the probe activation was found to be suppressed in the Camostat treated animals when compared to untreated animals (P<0.01 figure 4b). Unlike the edema ratio (figure 4a), the probe activation graph for corresponding pancreas showed that there was a significant difference in Camostat treated animals and controls animals (P<0.05). The sensitivity obtained from the *ex vivo* imaging is much higher than just the observation of pancreatic weight change.

#### 9.4 Application of the trypsin activatable mPEG-PL-Cy5.5 probe to test trypsin inhibitors

**Figure 5:**
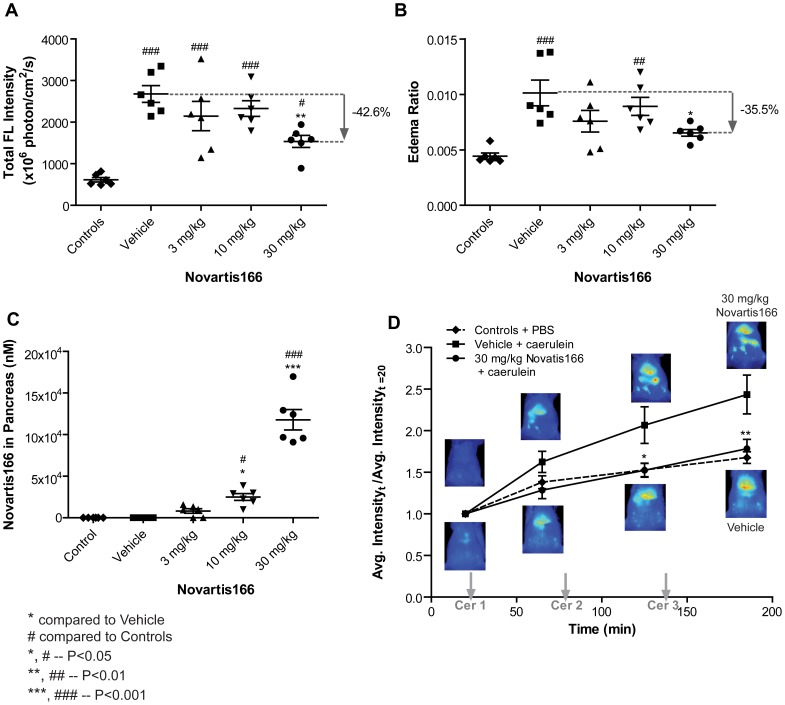
Application of the mPEG-PL-Cy5.5 probe to evaluate the efficacy of trypsin inhibitor Novartis166. Animals were administered PBS, Vehicle ( = “control”), or varying concentration of Novartis166 IP prior to caerulein induction of pancreatitis. Pancreas were excised and in vivo fluorescent images were acquired at the end of a 3 h caerulein induced pancreatitis study. A) The graph shows the fluorescent intensity of the mPEG-PL-Cy5.5 probe. Novartis166 at 30 mg/kg significantly reduced the activation of the trypsin mPEG-PL-Cy5.5 probe compared to other the Vehicle group. B) The corresponding edema ratio also indicated that Novartis166 at 30 mg/kg significantly abrogated the development of edema compared to the Vehicle group. C) The amount of Novartis166 present in the pancreas was also assessed. The amount of Novartis166 present correlated to the dose administered. D) At 30 mg/kg of Novartis166, in vivo probe activation time course was also evaluated. With increasing time, the activation of the mPEG-PL-Cy5.5 probe was significantly reduced. Data represented mean ± SEM.

We validated our capability to obtain a dose response relationship using *in vivo* imaging. An in-house trypsin inhibitor (Novartis166) was used. Novartis166 was administered intra peritonealy (IP) using a vehicle, one hour prior to caerulein administration, at doses (3, 10, or 30 mg/kg). Control animals that did not receive any caerulein were used for baseline comparison. As seen in figure 5a, at 30 mg/kg dose, there was a significant difference between vehicle+caerulein-treated baseline controls and trypsin inhibitor treated animals (P<0.01). However, the edema graph (figure 5b) indicated no significant difference between baseline controls and treated animals. In addition, we were able to gain a dose response relationship. At 3 and 10 mg/kg, there was no significant difference between untreated and treated animals (figure 5a). We further validated our capability to use *in vivo* imaging for observing the effect of a trypsin inhibitor in real time. As shown in figure 5d, at 30 mg/kg dose, the animals were not significantly different from baseline control animals in terms of the concentration of active trypsin in the pancreas. These results indicate that *in vivo* imaging may be used to predict the outcome of a trypsin inhibitor dynamically.

**Figure 6:**
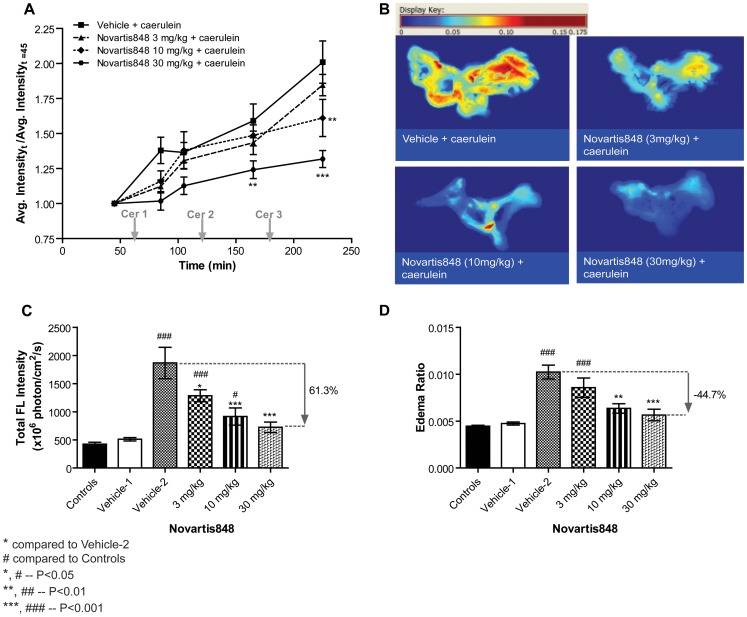
Evaluation of trypsin inhibitor Novartis848 on intrapancreatic trypsin activity and edema formation using mPEG-PL-Cy5.5 probe. Animals were administered PBS, vehicle, or varying concentration of Novartis848 IP 1 h prior to caerulein induction of pancreatitis (n = 5∼7). Vehicle-1 = vehicle for Novartis848+ SC PBS, Vehicle-2 = vehicle for Novartis848+ SC caerulein. A) Animals were administered the mPEG-PL-Cy5.5 probe to monitor the activity of trypsin in caerulein induced pancreatitis animals. Animals receiving 30 mg/kg of Novartis848 and caerulein showed marked reduction in the signal intensity in the areas of the pancreas compared to vehicle animals. B), C) & D) Pancreas were excised, weighed and fluorescent images were acquired at the end of a 3 h caerulein induced pancreatitis study. B) Fluorescence heat maps of excised pancreas. C) Excised pancreas fluorescence showed results similar to the *in vivo* imaging study. At 30 mg/kg of Novartis848 the fluorescence of the probe was significantly reduced in comparison to Vehicle-2 animals. (D) The edema ratio. Data represented mean ± SEM.

To establish a dose response relationship with real time *in vivo* imaging, we used another in-house trypsin inhibitor Novartis848. As shown in figure 6a, at 10 and 30 mg/kg doses of Novartis848, there was a significant difference between treated and untreated animals by the third dose of caerulein. Animals receiving 30 mg/kg of Novartis848 and caerulein showed marked reduction in the signal intensity in the areas of the pancreas compared to Vehicle-2 animals (P<0.01 and P<0.001 after Cer2 and Cer3 time points respectively). The difference was not apparent at other concentrations of Novartis848 indicating a dose effect on the activation of the mPEG-PL-Cy5.5 probe. These results validate that fluorescent whole body imaging can be used in an animal like rat to assess the trypsin-dependent edema formation in the absence and presence of a trypsin inhibitor in the pancreas. *In vivo* results were further validated by *ex vivo* examination of the pancreas. As shown in figure 6c, 3, 10 and 30 mg/kg of Novartis848 doses were significantly different from untreated Vehicle-2 animals (P<0.05, P<0.001, and P<0.001 respectively). However, the results were not the same on the edema ratio chart (figure 6d). Our studies indicate that *in vivo* imaging maybe used to assess the trypsin-dependent edema formation in the absence and presence of a trypsin inhibitor in a dose response manner.

## Discussion

In this report, we successfully demonstrated the use of an activatable fluorescent smart probe to image the trypsin-dependent development of experimental pancreatitis and treatment response to protease inhibitors in a caerulein-injection animal model. The development of optical fluorescence imaging in the near infrared has extended the field of *in vitro* fluorescence imaging at the molecular level to *in vivo* disease diagnosis and measurement of treatment response in animal models. Optical probes that emit in the near infrared can be employed successfully for various biomedical applications since hemoglobin and water have low coefficient of absorption in this region [23]. Therefore, deeper tissue can be accessed using fluorescent optical signal. Highly specific activatable, or “smart” [13] fluorescent probes have further enabled the real time visualization of the desired molecular function combined with specific localization.

In pancreatitis, the pre-mature activation of trypsinogen to trypsin leads to autodigestion of the pancreatic tissue [24], [25]. The use of a fluorescent probe activated upon cleavage by trypsin was attractive for the characterization of intrapancreatic trypsin inhibitors in an *in vivo* model of experimental pancreatitis. Our in-house developed mPEG-PL-Cy5.5 probe was very selectively cleaved by trypsin as confirmed by an assay panel of pancreatic proteases. Except for rat pancreatic elastase we cannot formally exclude that the selectivity profile of the probe based on rat orthologues of the pancreatic proteases differs from that of the tested human enzymes. However, this is considered very unlikely and irrelevant, because the concentration of trypsin in the pancreas of the caerulein-treated rats is reported to be much higher than other proteolytic enzymes.

Other available protease-activated fluorescent detectors are almost exclusively for *in vitro* studies. Commercially available tryptase sensors are based on substrates with fluorophores in the visible region. These wavelengths are not useful for *in vivo* optical imaging operating with fluorescence in the near-infrared region, required for better light penetration and lower scattering in tissue [23], [26]. In addition, protease substrates for *in vitro* studies are small peptides with a very unfavorable PK for *in vivo* studies. Several enzyme activated fluorescent probes have been previously fabricated for *in vivo* studies and examined for use in tumor models [27], [28], [29], [30], [31]. In contrast to the mostly passive accumulation of such probes in tumors of mouse models, we had to optimize biodistribution and PK/PD of the probe for adequate pancreatic exposure and found that the ideal probe size is in the 200 kD range. To our knowledge the work presented here is the first use of an enzyme activated fluorescent probe in the monitoring of an organ dysfunction and subsequent therapeutic evaluation. Macroscopic fluorescence imaging is used in mice, particularly nude mice, as lack of hair and translucent skin present minimum attenuation. However, this report is unique as we successfully demonstrate the use of optical imaging in a widely used rat model for pancreatitis. We provide evidence that the mPEG-PL-Cy5.5 probe was activated upon induction of pancreatitis and examining the abdominal cavity for the source of the fluorescent signal further validated the results. Pancreatitis induction with repeated caerulein administration is commonly used in animal models and is accompanied by edema development in the pancreas within one hour [32], [33], [34]. To validate the increase in mPEG-PL-Cy5.5 probe activation and accumulation with time, we used repeated caerulein administration. The subsequent activation and increased fluorescence specific to the pancreatic region mapped trypsin activity in the pancreas. As expected, we also observed probe activation in the liver due to metabolism. Currently, it is difficult to investigate details of ongoing enzymatic events *in vivo* [24]. The use of an activatable probe helps overcome these limitations and the onset of enzyme activation and its correlation to disease markers can be examined in real time, if validated with excised tissue and biochemical correlation as shown in this report. When the pancreas was pre-protected from caerulein disease induction by administration of the trypsin inhibitor Camostat, mPEG-PL-Cy5.5 probe activation was highly suppressed. We successfully used this technique to evaluate the extent of dose dependent trypsin inhibition by other selective trypsin inhibitors. Reduction in edema is one of the anatomical outcome measures used to evaluate the effectiveness of potential therapies against experimental pancreatitis in rodent models [35], [36], [37]. In the current study, we observed that the edema ratio readout did not always compare quantitatively to the corresponding fluorescence recorded from pancreas *ex vivo.* Although the edema graph (figure 4) indicates that treatment with Camostat results in no significant difference compared to normal animals (controls), the total fluorescence graph shows that treatment with Camostat lowered the active trypsin, but there was still a significant difference compared to control animals. Similarly, treatment with 30 mg/kg of Novartis166 (figure 5) showed no significant difference compared to controls on the edema graph, and vice versa on the fluorescence intensity graph. We, therefore, conclude that edema reduction is not an accurate indicator of trypsin inhibition in the pancreas. A biodistribution analysis of trypsin from explanted pancreas (figure 4d) indicated a high level of variability between caerulein treated and untreated control animals making this assessment less reliable measure of outcome. Therefore, a real time assessment of function, as in our case with a trypsin activatable probe, provides a more reliable tool for effectiveness of a trypsin inhibitor. Nevertheless, there are certain limitations to this method. The permeability of the activatable probe into the target tissue may be an important player in the assessment of disease severity. However, even in the three-hour model we never observed a plateau of the fluorescence signal, typically a clear indication of limited probe availability. In this study, we established a dose response relationship between protease inhibitor, intrapancreatic protease inhibition and edema formation, respectively. Our results indicated that we were able to establish the effect of trypsin inhibitors in real time. Therefore, the trypsin activatable mPEG-PL-Cy5.5 probe could be used as a dynamic mode-of-action biomarker for trypsin activity in a preclinical model of experimental pancreatitis. The *in vivo* imaging enables correlating end point readouts, but is a reliable method to assess the effects dynamically.

The ability to image pancreas in the rat model with the use of a blood pool fluorescent marker to trace the development of edema *in vivo* was also confirmed. In theory, by doing so, one can conclusively demarcate the pancreas in real time as the disease progresses and correlate the fluorescent signal from the activated probe channel to that from the blood pool contrast agent to dynamically measure trypsin activity in the target tissue. Two fluorescent probes were co-administered (protease activatable mPEG-PL-Cy5.5 probe labeled with Cy5.5-670/690 and a blood pool probe, 750/780). The results of these combined studies were inconsistent with the data we observed from individual experiments. We observed that the fluorescent intensity of the blood pool agent was significantly higher in the liver than in a blood pool probe only experiment. We were able to establish these anomalies because of the powerful spectral unmixing capabilities. Although, in this report there was limited success in combining the blood pool contrast agent and the activatable mPEG-PL-Cy5.5 probe in the same animal simultaneously, we were able to identify the underlying problem. When co-administered, upon activation of the mPEG-PL-Cy5.5 probe, the shape of the emission spectrum of the Cy5.5 dye overlapped with the emission of the blood pool probe. Therefore, when used in the same animal, unmixing the two channels became difficult giving false indications. Although an activatable probe was fabricated with Cy5.5 dye, the emission fluorescence leg significantly broadened to overlap with the emission of the blood pool fluorescent agent. Further optimization of the choice of fluorophores and the respective emission bandwidth may help in successful unmixing of the two channels. By using data that permitted real spectral unmixing [38] we were able to select for fluorescence resulting from activation of the mPEG-PL-Cy5.5 probe. Typically only fluorescence is collected with the use of fixed range filter sets reporting broad readings of pooled fluorescence. Such readings may falsely indicate fluorescence signal from a particular fluorophore in another channel when two probes are used in the same animal. Therefore, careful consideration should be given to the choice of the *in vivo* fluorescence imager [38], [39], [40]. In order to overcome unmixing complexities, alternative dyes such as Alexa fluorophore dyes that are known to have narrower emissions bands compared to cyanines could be used, in combination with further separated wavelength fluorophores.

The development of activatable probes specific to different enzymes can further enable identification and better understanding of the underlying cause of pancreatitis. It is known that autodigestion due to premature trypsinogen activation leads to pancreatic failure. However, little is known about the role of other proteases as precursors of such an event [41], [42], [43]. Cathepsin B is one such candidate that could be responsible for triggering the sequence of events that leads to pancreatitis [41]. In our studies, we observed that there was low level activation of the mPEG-PL-Cy5.5 probe by cathepsin B enzyme indicated by low fluorescence signal for cathepsin B in the enzyme panel assay for probe activation (figure 1). Moreover, the two tested trypsin inhibitors Novartis166 and Novartis848 do not inhibit cathepsin B at all. Therefore, we examined the effect of a cathepsin B inhibitor on mPEG-PL-Cy5.5 probe activation in the rat caerulein-injection model. In the presence of cathepsin B inhibitor (Novartis242), mPEG-PL-Cy5.5 probe activation was subdued (figure S3, P<0.05) indicating that cathepsin B may have a role in trypsin activation in the pancreas. As expected, there was no effect of the combination of the trypsin inhibitor Novartis166 with Novartis242 and the outcomes were similar to treatment with Novartis166 alone indicating that Novatis166 mode-of-action is only by trypsin inhibition.

In conclusion, we established a mode-of-action biomarker assay for intrapancreatic trypsin activity in a preclinical model of experimental pancreatitis and characterized protease inhibitors in this model. This model could be applied to preclinically optimize trypsin inhibitors towards target inhibition in the target tissue. Since fluorescent probes and optical imaging technolgies have already been used in patients, a possible translation into the clinic could be envisioned.

## Supporting Information

Figure S1 **Measurement of pH sensitivity of the trypsin activatable mPEG-PL-Cy5.5 imaging probe.** A) pH sensitive rat Trypsin cleavage of 2 different fluorescence intensity substrates (mPEG-PL-Cy5.5 imaging probe vs GPR-Rh110 substrate). Although a pH dependent decrease in enzymatic activity is observed, it is independent of the substrate used. B) pH sensitivity of mPEG-PL-Cy5.5 in the presence and absence of rat Trypsin. Enzyme kinetic is measured using rat Trypsin and the Cy5.5 imaging probe at 3 different pH values. The background signal (substrate only) remains stable whereas only the enzymatic activities differ suggesting that differences derive from different enzymatic activities at different pH values, not from the substrate itself.
(TIF)

Figure S2 **Examination of cellular internalization of the trypsin activatable probe in Mia PaCa cells and Macrophages.** A) For doxycycline (dox) mediated trypsin activation; cell media was replaced with phenol free media +/− dox, 10 µM,+GPR-Rh110, +0.1 µM mPEG-PL-Cy5.5 probe,+antifade and imaged. B) For trypsin activation with caerulein, cell media was replaced with phenol free media +/−40 nM caerulein, 10 µM,+Rh-substrate, +0.1 µM mPEG-PL-Cy5.5,+antifade and imaged. All exposure times were kept constant for comparison. Images shown are in individual fluorescence channel overlaid with phase to demarcate cells. C) Macrophages were incubated with 10 µl (2 mg/ml mPEG-PL-Cy5.5probe) +2 ml (phenol-free DMEM+10% FBS) Cells were imaged at 20 minutes after incubation. Images shown are with and without phase and overlay to demarcate cells.
(TIF)

Figure S3 **Effect of cathepsin B inhibitor Novartis242 examined by mPEG-PL-Cy5.5 probe.** A) Real time probe activation in caerulein-injection model with or without the administration of the cathepsin inhibitor Novartis242. It was observed that by the 3^rd^ caerulein injection, at 10 mg/kg of Novartis242, a significant difference (*P<0.05) was observed compared to the vehicle animals. The combination of trypsin inhibitor Novartis166 at 30 mg/kg with 10 mg/kg of Novartis242 showed significantly better reduction in probe activation at caerulein 2 and 3 (**P<0.01, ***P<0.001) compared to vehicle. B) *Ex vivo* examination of the pancreas revealed that there was no effect of dose from Novartis242 upon trypsin probe activation. The combination treatment, when compared to individual treatment with Novartis166 at 30 mg/kg did not indicate any difference. However, both treatments showed significantly better reduction in active trypsin. Data represented mean ± SEM. * compared to vehicle. # compared to controls.
(TIF)

Material S1 **Additional Methods and Results.**
(DOCX)
