# Correction: *In Vivo* Imaging with Fluorescent Smart Probes to Assess Treatment Strategies for Acute Pancreatitis

**DOI:** 10.1371/annotation/e3c6b1fa-9bba-418a-950e-1082d7a6e30a

**Published:** 2013-10-10

**Authors:** Abhiruchi Agarwal, Andreas Boettcher, Rainer Kneuer, Farid Sari-Sarraf, Adriana Donovan, Julian Woelcke, Oliver Simic, Trixi Brandl, Thomas Krucker

Figure S3 is a duplicate of Figure S1. Please view the correct Figure S3 here: http://plosone.org/corrections/pone.0055959.s003.cn.tif. The figure legend is correct. 

