# Supplementary figures and images for: In Vivo Imaging with Fluorescent Smart Probes to Assess Treatment Strategies for Acute Pancreatitis
Source: PLoS One. 2013 Feb 11;8(2):e55959. doi: 10.1371/journal.pone.0055959 (PMC3569412; doi:10.1371/journal.pone.0055959)

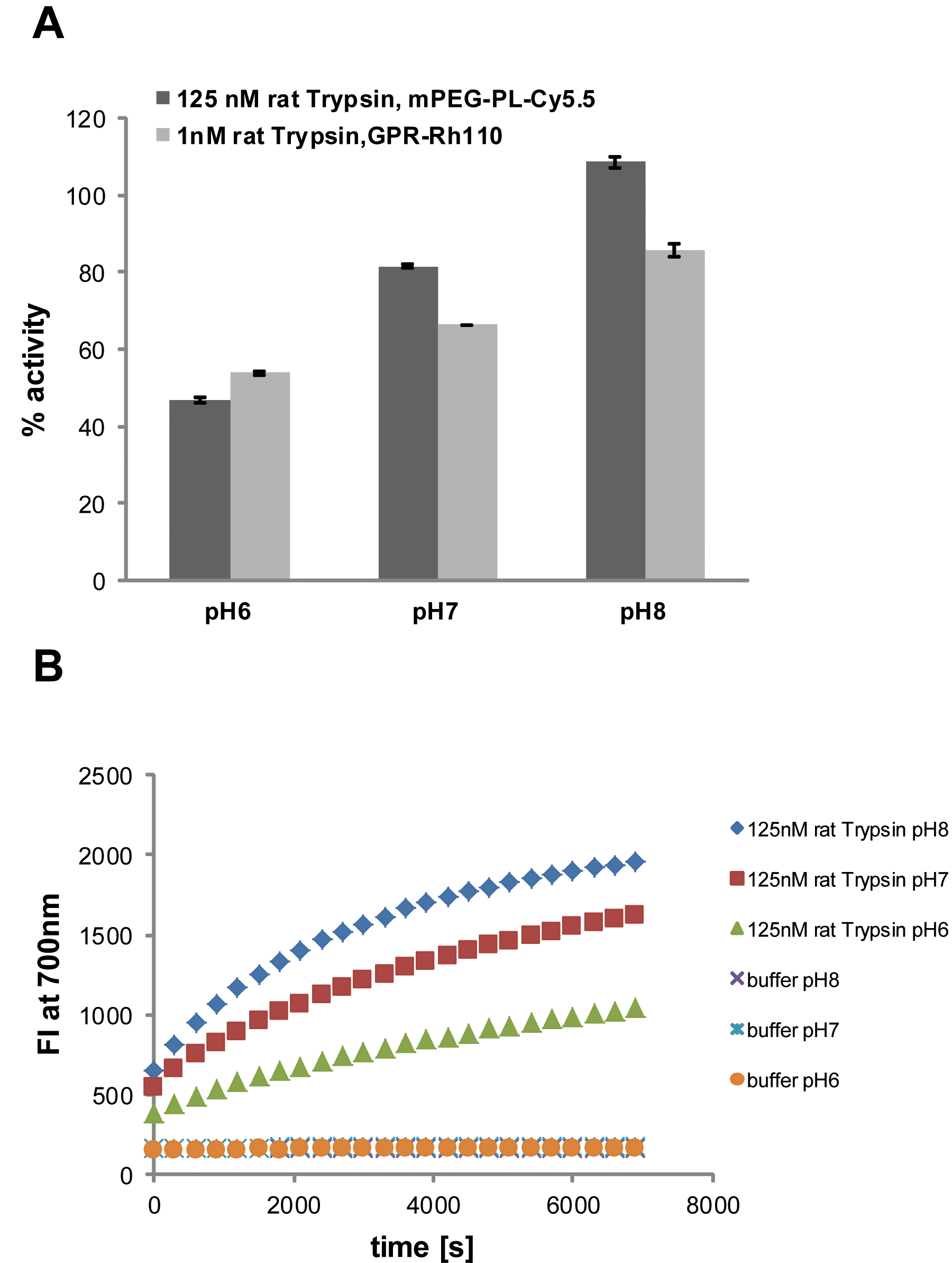

Supplement: Figure S1 — Measurement of pH sensitivity of the trypsin activatable mPEG-PL-Cy5.5 imaging probe. A) pH sensitive rat Trypsin cleavage of 2 different fluorescence intensity substrates (mPEG-PL-Cy5.5 imaging probe vs GPR-Rh110 substrate). Although a pH dependent decrease in enzymatic activity is observed, it is independent of the substrate used. B) pH sensitivity of mPEG-PL-Cy5.5 in the presence and absence of rat Trypsin. Enzyme kinetic is measured using rat Trypsin and the Cy5.5 imaging probe at 3 different pH values. The background signal (substrate only) remains stable whereas only the enzymatic activities differ suggesting that differences derive from different enzymatic activities at different pH values, not from the substrate itself. (TIF) [file pone.0055959.s001.tif]

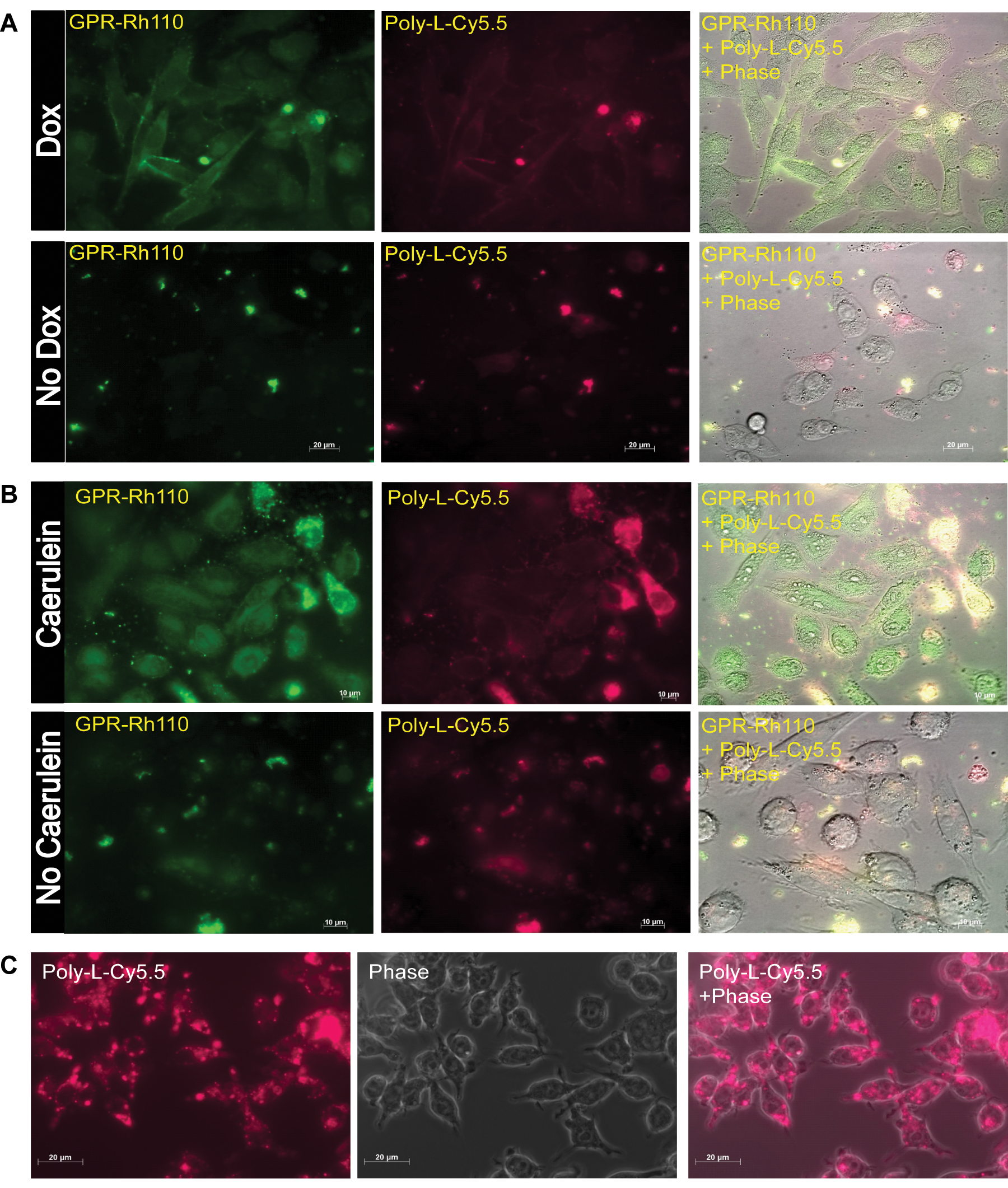

Supplement: Figure S2 — Examination of cellular internalization of the trypsin activatable probe in Mia PaCa cells and Macrophages. A) For doxycycline (dox) mediated trypsin activation; cell media was replaced with phenol free media +/− dox, 10 µM,+GPR-Rh110, +0.1 µM mPEG-PL-Cy5.5 probe,+antifade and imaged. B) For trypsin activation with caerulein, cell media was replaced with phenol free media +/−40 nM caerulein, 10 µM,+Rh-substrate, +0.1 µM mPEG-PL-Cy5.5,+antifade and imaged. All exposure times were kept constant for comparison. Images shown are in individual fluorescence channel overlaid with phase to demarcate cells. C) Macrophages were incubated with 10 µl (2 mg/ml mPEG-PL-Cy5.5probe) +2 ml (phenol-free DMEM+10% FBS) Cells were imaged at 20 minutes after incubation. Images shown are with and without phase and overlay to demarcate cells. (TIF) [file pone.0055959.s002.tif]

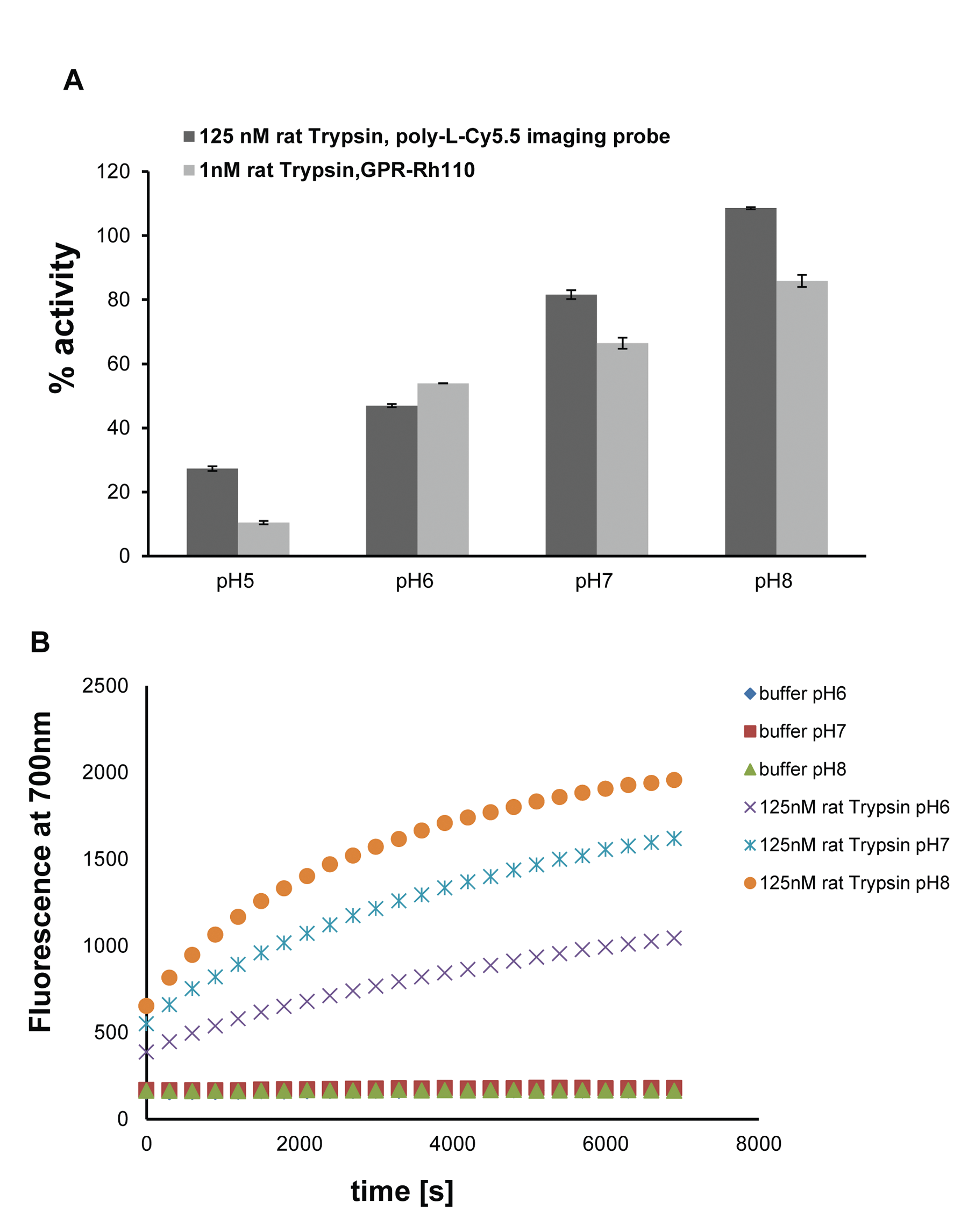

Supplement: Figure S3 — Effect of cathepsin B inhibitor Novartis242 examined by mPEG-PL-Cy5.5 probe. A) Real time probe activation in caerulein-injection model with or without the administration of the cathepsin inhibitor Novartis242. It was observed that by the 3rd caerulein injection, at 10 mg/kg of Novartis242, a significant difference (*P<0.05) was observed compared to the vehicle animals. The combination of trypsin inhibitor Novartis166 at 30 mg/kg with 10 mg/kg of Novartis242 showed significantly better reduction in probe activation at caerulein 2 and 3 (**P<0.01, ***P<0.001) compared to vehicle. B) Ex vivo examination of the pancreas revealed that there was no effect of dose from Novartis242 upon trypsin probe activation. The combination treatment, when compared to individual treatment with Novartis166 at 30 mg/kg did not indicate any difference. However, both treatments showed significantly better reduction in active trypsin. Data represented mean ± SEM. * compared to vehicle. # compared to controls. (TIF) [file pone.0055959.s003.tif]
