# Supplementary material for: In Vivo Imaging with Fluorescent Smart Probes to Assess Treatment Strategies for Acute Pancreatitis
Source: PLoS One. 2013 Feb 11;8(2):e55959. doi: 10.1371/journal.pone.0055959 (PMC3569412; doi:10.1371/journal.pone.0055959)
Supplement: Material S1 — Additional Methods and Results. (DOCX) [file pone.0055959.s004.docx]

**Supplemental Material**

# Additional Methods & Results

## Effect of pH on mPEG-PL-Cy5.5 probe’s sensitivity to trypsin cleavage

We determined the pH effect on the performance of our probe by testing trypsin activity and substrate response at pH ranging from 6 to 8. All enzyme assays were conducted in 384-well plates with a final substrate (mPEG-PL-Cy5.5 probe or GPR-Rh110) concentration of 0.2 μM diluted in the appropriate assay buffer at various pH (6, 7 and 8) in a final assay volume of 25 μl. Substrate was added to rat trypsin and incubated for 1h at 37°C prior to measurement or for enzyme kinetics as indicated in Figure S1.

Plate measurements were conducted by the means of a Safire2 microplate reader (TECAN, Maennedorf, Switzerland) and wavelengths of 680 nm and 700 nm were taken for fluorescence excitation and emission acquisition, respectively. The bandwidths were set to 10 nm in both the excitation and the emission path. Three flashes per measurement sufficiently excited the fluorescence in each.

We found that, not unexpectedly, the enzyme activity decreases with lower pH. However, as shown in Figure S1, it is independent of the substrate sequence, as the cleavage of the standard GPR-Rh110 substrate at different pH parallels with the probe, and therefore is not of concern. The substrate alone is equally stable at all three tested pH (6, 7 and 8) (Figure S1). The enzyme activity on the probe decreases with lower pH, however, it is still very high and only a factor of 2 lower at pH=6 than at pH=8.

## *In vitro* activation and localization of mPEG-PL-Cy5.5 probes: intra- vs. extra-cellular

To examine if the probe fluorescence observed in *in vivo* imaging is detecting trypsin within or outside the cell, we used caerulein stimulated Mia PaCa cells and observed the activation of the mPEG-PL-Cy5.5 probe. The human pancreatic undifferentiated cell line Mia PaCa maintains many characteristics of normal pancreatic acinar cells and is widely used as an “in vitro” model to study the exocrine pancreas.

Mia PaCa cells (ATCC) were seeded sparingly on glass coverslips and cultured overnight. The next day, for doxycycline (dox) mediated trypsin activation; media was replaced with serum free media with or without dox and cultured for another 24 hours. For trypsin activation with dox, cell media was replaced with phenol free media +/- dox, 10µM, + GPR-Rh110, + 0.1µM mPEG-PL-Cy5.5 probe, + antifade and imaged. For trypsin activation with caerulein, cell media was replaced with phenol free media +/- 40nM caerulein, 10µM, + Rh-substrate, + 0.1µM mPEG-PL-Cy5.5 probe, + antifade and imaged. All exposure times were kept constant for comparison.

Raw macrophages were cultured sparingly on glass coverslips overnight. On the day of imaging, macrophages were washed with PBS. 10 µl (2mg/ml mPEG-PL-Cy5.5probe) + 2 ml (phenol-free DMEM+10% FBS) was added to cells. Cells were imaged at 20 minutes after incubation (AxioObserver Z1, Carl Zeiss, Thornwood, NY).

Experiments with cultured Mia PaCa cells (activated trypsin either with Caerulein or +/- Doxycycline control, Figure S2) show that activated probe can be detected inside cells, in particular more prominently in activated cells. Another experiment with macrophages shows activated probe inside cells suggesting the involvement of endocytosis. In addition, it is nearly impossible under these conditions to absolutely quantify the extracellular fluorescent signal because of the large volume and constant dilution. Based on these *in vitro* results and the size of the large size of the mPEG-PL-Cy5.5 probe, we feel safe to conclude that the probe is most likely present in the edematous fluid in the pancreas and that the vast majority of the fluorescent signal detected *in vivo* is coming from probe activated outside the acinar cells (Also see Sherwood MW Proc Natl Acad Sci USA 104: 5674-5679, 2007).
